# Thermophysical and Chemical Evaluation of Macaw Palm Almond Oil as a Biobased Heat-Transfer Fluid Candidate for Medium-Temperature Solar Thermal Applications

**DOI:** 10.1021/acsomega.6c06774

**Published:** 2026-09-17

**Authors:** Markus AO Porangaba, Thaís Meira Menezes, Isabella Colatino de Lima Veiga, Natalia Angelita Albuquerque de Melo, Lucas Meili, Julio Inacio Holanda Tavares Neto, Amanda Santana Peiter, Leonardo Faustino Lacerda de Souza

**Affiliations:** † Engineering and Agricultural Sciences Campus (CECA), 28112 Federal University of Alagoas (UFA), Rio Largo, Alagoas 57100-000, Brazil; ‡ Cabo de Santo Agostinho Academic Unit (UACSA), Federal Rural University of Pernambuco (UFRPE), Cabo de Santo Agostinho, Pernambuco 54518-430, Brazil; § Laboratory of Processes Applied to Water Treatment, Center of Technology, Federal University of Alagoas, Av. Lourival Melo Mota, s/n, Campus A.C. Simões, Tabuleiro do Martins, Maceió, Alagoas 57072-970, Brazil

## Abstract

Macaw palm (*Acrocomia aculeata*) almond oil was evaluated as a potential renewable heat-transfer fluid (HTF) candidate for medium-temperature solar thermal applications through thermophysical and chemical characterization. The oil was extracted using Soxhlet extraction and characterized by gas chromatography with flame ionization detection (GC-FID), Fourier transform infrared spectroscopy (FTIR), ultraviolet–visible spectroscopy (UV–Vis), thermogravimetric analysis (TGA), and differential scanning calorimetry (DSC). The extraction yield was 15.74 ± 2.85% (w/w), with an acid value of 3.44 ± 0.02 mg KOH·g^–1^, density of 0.917 ± 0.006 g·cm^–3^ at 20 °C, and kinematic viscosity of 29.75 ± 0.76 mm^2^·s^–1^ at 40 °C. Fatty acid composition revealed a predominance of lauric acid (39.36%), followed by oleic (29.99%) and linoleic acids (14.47%), indicating a mixed saturated-unsaturated profile with implications for viscosity behavior and oxidative susceptibility. FTIR and UV–Vis analyses confirmed the triglyceride structure and indicated early oxidation markers associated with unsaturated lipid fractions. Thermal analysis indicated the onset of measurable degradation around 300 °C, suggesting that continuous thermal-fluid operation should be maintained below this temperature. Compared with other vegetable oils previously investigated for thermal-fluid applications, macaw palm almond oil presents favorable density and viscosity characteristics, although oxidative stability and acid value remain engineering concerns requiring pretreatment or formulation improvements. These findings represent a first-stage screening assessment of macaw palm almond oil as a biobased HTF candidate and support further investigation involving oxidative aging, thermal cycling, corrosion compatibility, and pilot-scale validation.

## Graphical abstract

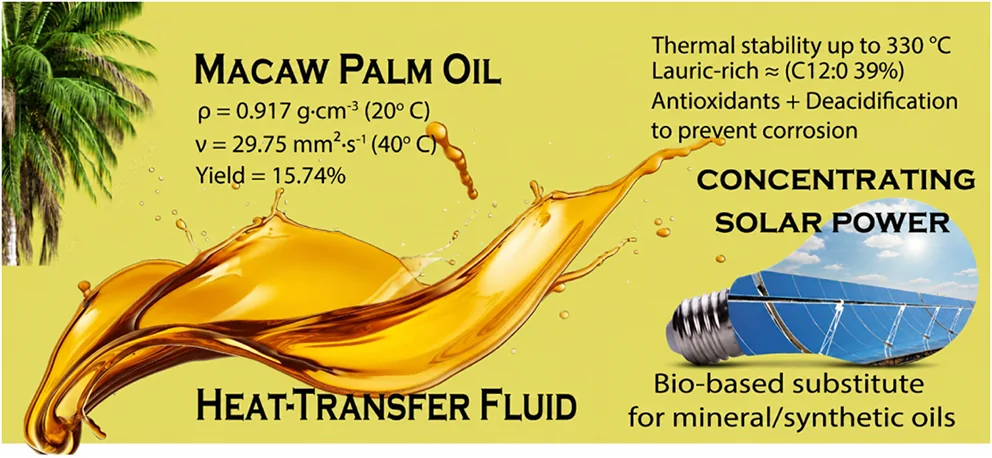

## 1 Introduction

Concentrating solar power (CSP) has emerged as a promising technology for decarbonizing the power sector because, unlike most intermittent renewable energy systems, it can be directly integrated with large-scale thermal energy storage (TES), enabling dispatchable electricity generation and operational flexibility. In addition to grid-scale electricity production, solar thermal technologies—including CSP systems—have applications in industrial process heating, standalone thermal storage, desalination, and fuel or chemical production.^1^ However, the broader deployment of these technologies remains strongly dependent on the performance, stability, and economic viability of heat-transfer fluids (HTFs) and thermal energy storage materials (TESMs).

Conventional HTFs, particularly synthetic thermal oils, remain commercially dominant but present important technical and environmental limitations, including relatively low thermal decomposition temperatures, limited density, high flammability, elevated vapor pressure, toxicity concerns, and high operational costs.^2,3^ These limitations reinforce the need for renewable, safer, and environmentally compatible alternatives.

It is important to distinguish concentrating solar power (CSP) from the broader category of solar thermal applications. While CSP specifically refers to systems that use concentrated solar irradiation to generate high-temperature thermal energy for electricity production via thermodynamic cycles, solar thermal technologies encompass a wider range of heat-based applications, including industrial process heating, desalination, solar cooling, domestic hot water generation, and standalone thermal storage systems. This distinction is relevant because the thermophysical and thermal stability requirements for heat-transfer fluids vary substantially depending on the target operating temperature and application.^4^

Vegetable oils have attracted increasing attention as potential HTF candidates due to their renewable origin, biodegradability, lower toxicity, and broad availability as locally sourced feedstocks. Recent studies have emphasized that the selection of heat-transfer fluids remains one of the most critical design parameters in solar thermal systems because fluid properties directly affect thermal efficiency, pumping requirements, heat exchanger performance, operational safety, and overall economic feasibility.^5,6^

In countries such as Brazil, favorable agro-climatic conditions may further improve their economic competitiveness compared with imported mineral or synthetic thermal fluids.^7^ From a technical perspective, vegetable oils may offer advantageous properties such as high flash points, low volatility, good lubricity, and elevated viscosity indices. However, their application in thermal systems remains limited by challenges associated with oxidation, thermal degradation, viscosity increase under prolonged heating, and material compatibility. Several vegetable oils, including soybean, palm, and jatropha oils, have previously been investigated as potential thermal fluids, highlighting both the promise and limitations of this class of renewable materials. Recent research has increasingly explored environmentally sustainable alternatives to conventional petroleum-derived HTFs, driven by lifecycle sustainability concerns and stricter environmental performance requirements. However, oxidative stability and viscosity behavior at elevated temperatures remain major technical barriers to the practical implementation of biobased fluids.^5^

Within this context, *Acrocomia aculeata* (macaw palm) represents an interesting but still underexplored feedstock. This native Brazilian palm is widely distributed across subtropical and semiarid regions, exhibits strong ecological adaptability, and offers attractive oil productivity, reaching approximately 4,000 kg oil·ha^–1^ under favorable conditions.^8,9^ In addition, macaw oil presents a fatty acid composition that may be relevant for thermal applications, particularly due to its significant content of saturated and monounsaturated components, which can influence both fluidity and thermal stability.^10^

Despite this potential, the applicability of macaw palm oil as a heat-transfer fluid remains insufficiently investigated, particularly regarding its thermophysical and thermal behavior in the context of solar thermal systems. To the best of current knowledge, no study has specifically evaluated *Acrocomia aculeata* almond oil from the perspective of its applicability as a renewable heat-transfer fluid for solar thermal systems. Existing studies involving this feedstock are predominantly focused on biodiesel production, food applications, or general physicochemical characterization rather than engineering-oriented thermal fluid assessment. This establishes a clear research gap regarding its thermophysical suitability for renewable thermal energy applications.

Therefore, this study presents a first-stage screening assessment of macaw (*Acrocomia aculeata*) almond oil as a potential renewable HTF candidate through physicochemical, spectroscopic, and thermophysical characterization. Specifically, acid value, density, and kinematic viscosity were determined; fatty acid composition was evaluated by GC-FID; molecular structural features and oxidation-related markers were assessed using FTIR and UV–Vis spectroscopy; and thermal behavior was investigated through TGA and DSC analyses. Unlike studies that focus solely on compositional characterization, this work aims to interpret these properties from an HTF applicability perspective, identifying both the opportunities and engineering limitations associated with this renewable material. Although operational HTF validation, thermal cycling performance, and techno-economic assessment remain beyond the scope of this study, the findings contribute to expanding the portfolio of renewable candidate fluids for sustainable solar thermal applications.

## 2 Experimental Methods

### 2.1 Materials

The reagents used for macauba oil extraction included Hexane (100%, Synth, Brazil) and Anhydrous Sodium Sulfate P.A. (99%, Neon, Brazil), both of which were used as received without any further treatment. All apparatus cleaning procedures were performed using deionized water.

### 2.2 Oil Preparation and Extraction

Macaw palm (*Acrocomia aculeata*) fruits were collected at the Agricultural Sciences Engineering Campus (CECA) of the Federal University of Alagoas (UFAL), Brazil, at different maturation stages (green, intermediate, and ripe). After collection, the fruits were cleaned, peeled, and subjected to drying in a forced-air oven (Solab, model SL-100, 15,000 W) at 50 °C for approximately 72 h to reduce moisture content.

Following drying, the almond material was manually separated using a stainless-steel knife, crushed using a grain grinder (Hamilton Beach, model 80393BZ220, 150 W), and maintained under controlled drying conditions at 50 °C until extraction.

Oil extraction was performed using the Soxhlet method, selected due to its high extraction efficiency for lipid recovery from oleaginous matrices. Three extraction cartridges were prepared using filter paper and filled with previously weighed crushed almond samples.

The extraction system consisted of three 1 L round-bottom flasks connected to Soxhlet extractors and condensers. In each extraction unit, 125 mL of hexane was used as the solvent for approximately 17 g of macauba palm almond, resulting in a solvent-to-sample ratio of approximately 1:7.3 (m/v). The extraction process was conducted for approximately 5 h at about 70 °C using heating mantles.

Temperature monitoring during extraction was performed using a digital thermometer (Incoterm, model 9226.16.0.00) to maintain appropriate solvent reflux conditions.

After extraction, the solvent was removed using a rotary evaporator (Büchi, model R-300, 100 W) at 60 °C under controlled vacuum for approximately 10 min.

Residual moisture was removed by adding anhydrous sodium sulfate (Na_2_SO_4_, 99%).

The extracted oil was stored in 50 mL amber glass containers protected from light with aluminum foil and kept at −20 °C until characterization.

### 2.3 Physical Characterization

#### 2.3.1 Extraction Yield

Initially, the oil extracted yield was determined by the difference between the initial and final mass after processing, expressed as a percentage according to eq 1:

1 $$Yield\;\left(\%\right)=\left(\frac{m_{1}-m_{2}}{m_{1}-m}\right)\times 100$$

Where *m* is the mass of the cartridge filter paper; *m*_1_ is the mass of the cartridge filter paper plus the mass of the sample before the extraction process; *m*_2_ is the mass of the cartridge filter paper plus sample after extraction. The weighing of *m*_2_ occurred after excess hexane evaporation in a ventilated oven at a temperature of 60 °C for 24 h.

#### 2.3.2 Acid Value Determination

The acid value (AV), a chemical parameter that relates to its nature and the quality of the product and its raw material, was determined to evaluate the oil’s conservation conditions. This index regards the decomposition of glycerides, accelerated by heating and light, forming free fatty acids^11^

The acid value was determined according to the standard method AOCS Cd 3d–63, approximately 2 g of oil sample was weighed into an Erlenmeyer flask, followed by addition of 25 mL of neutralized ethyl ether–ethanol solution (2:1 v/v) and two drops of 1% phenolphthalein indicator.

The mixture was titrated with standardized 0.01 N sodium hydroxide (NaOH) until appearance of a persistent pale pink end point.

The acid value was calculated according to eq 2:

2 $$AV=\frac{V\times f\times M\times 28.2}{m}$$

Where *AV* is the acid value in mg KOH·g^–1^; *V* is the volume of sodium hydroxide solution in mL used at title; *f* is the sodium hydroxide solution factor; *M* is the molarity of sodium hydroxide; *m* is the mass of the sample in g.

#### 2.3.3 Kinematic Viscosity

Kinematic viscosity was determined at 40 °C using a capillary viscometer setup under thermostatic control.

Approximately 8 mL of sample was introduced into the instrument. Flow time between calibrated meniscus marks was measured using a stopwatch.

Viscosity was calculated according to eq 3:

3 υ=t×K

Where υ is the kinematic viscosity in cst; *t* is the average of the times in seconds; *K* is the viscometer constant (K *=* 0.01509 mm^2^/s).

#### 2.3.4 Density Measurement

A digital densimeter Anton Paar model DMA 35 N measured the density. The oil (in a beaker) was kept in an ice water bath at a temperature of 20 °C, according to the ASTM D7777 standard. The analysis was carried out in triplicate and the arithmetic mean of the observed values was used, specifically as a reference physical characterization.

#### 2.3.5 Statistical and Uncertainty Considerations

All repeatable measurements were performed in triplicate, and the results are reported as mean ± standard deviation. This approach was used to account for experimental variability and to provide an estimate of the repeatability of the measurements.

For calculated properties, such as kinematic viscosity and acid value, the variability reported reflects the dispersion observed among the replicate determinations. Instrumental specifications and the controlled experimental conditions were also considered when assessing the reliability of the measurements.

As the present study was designed as a preliminary screening of macaw palm almond oil as a potential heat-transfer fluid, a formal propagation of uncertainty for each individual variable involved in the calculations was not performed. Nevertheless, the use of replicate measurements and the corresponding standard deviations provides an appropriate indication of experimental variability for the comparative and exploratory purposes of this study. A more detailed metrological uncertainty analysis should be included in future validation studies aimed at operational qualification of the fluid.

### 2.4 Chemical Characterization

#### 2.4.1 Fatty Acid Composition (GC-FID)

The identification and quantification of macaw oil compounds (including fatty acids) were determined using the Gas Chromatography analysis technique based on the separation of constituents between the stationary and mobile phases. The analysis employed a Varian CP-3900 CG chromatograph, model BP20 capillary column (15 m × 25 mm) (SGE), with a 20 M carbowax column (polyethylene glycol) as the stationary phase, and a flame ionization detector (FID), whose operating temperature is 280 °C. The initial temperature of 100 °C was used for 3 min; from 100 to 150 °C with a ramp of 50 °C/min; from 150 to 180 °C with a ramp of 1 °C/min; from 180 to 200 °C with a ramp of 25 °C, and a final temperature of 200 °C for 10 min. A sample of 1 μL was injected into an injector heated to 250 °C operated in 1:50 flow division mode.

#### 2.4.2 UV–Visible Spectroscopy

UV–Vis spectroscopy was used to investigate electronic absorption behavior and possible oxidation-related chromophoric species.

Analyses were performed using a double-beam UV–Vis spectrophotometer (Even, model EV-ESPECDF-BI) with wavelength range: 190–1100 nm; absorbance range: −300 to 3000 au; quartz/glass cuvettes with 1 mm optical path length.

#### 2.4.3 Fourier Transform Infrared Spectroscopy (FTIR)

FTIR analysis was performed to identify functional groups and molecular structural features.

Measurements were conducted using a Shimadzu IRTracer-100 spectrometer over the spectral range of 600–4000 cm^–1^, in attenuated total reflectance (ATR) mode, with a spectral resolution of 4 cm^–1^ and 32 accumulated scans.

This analysis was used to identify characteristic triglyceride absorptions and oxidation-related molecular signatures.

### 2.5 Thermal Characterization

#### 2.5.1 Thermogravimetric Analysis (TGA)

Thermal decomposition behavior was evaluated using thermogravimetric analysis. Analyses were performed using a Mettler Toledo TGA2 instrument under the following conditions: sample mass 5 ± 0.5 mg; aluminum crucibles; airflow of 60 mL·min^–1^; heating rate of 20 °C·min^–1^; temperature range of 30–600 °C.

#### 2.5.2 Differential Scanning Calorimetry (DSC)

The Differential Scanning Calorimetry (DSC) technique was used to determine the specific heat of macaw palm almond oil, whose characterization is based on the energy required to raise the temperature of the unit mass of a given substance by 1 K.^7,12^ Measurements were performed using a Mettler Toledo STAR System DSC instrument under: sample mass 5 ± 0.5 mg; aluminum crucibles; airflow of 60 mL·min^–1^; heating rate of 10 °C·min^–1^; temperature range of 20–250 °C. The DSC analysis was used to evaluate thermal events relevant to the thermal behavior of the oil.

## 3 Results and Discussion

### 3.1 Physical Properties of Macaw Palm Almond Oil

The extraction of macaw oil in this study yielded 15.74 ± 2.85% (w/w)—a value that is consistent with the literature and sits between the 6.1% reported by Amaral^13^ and the 16.5% reported by Hiane et al.,^14^ while remaining below the upper bound cited for dried fruits (∼25.5%). Such dispersion is expected and can stem from both biological and process determinants. Biologically, yield is modulated by genotype/ecotype, fruit maturity stage at harvest, and postharvest metabolism (e.g., endogenous lipase activity), all of which are further conditioned by environmental drivers that regulate plant productivity—particularly soil fertility and nutrient status, temperature regimes, water availability, and photoperiod.^13^ From a processing standpoint, reported values are sensitive to the moisture content and drying history of the feedstock, particle-size distribution after milling, solvent-to-solid ratio, extraction time and temperature, and the efficiency of solvent removal during concentration and storage. In this context, our result (obtained from a total extract volume of ∼30 mL) represents a realistic midrange performance for macaw almonds under the specified conditions, and it underscores the importance of clearly stating the mass basis (wet vs dry) and operational parameters when comparing extraction yields across studies. From an engineering perspective, extraction yield is not a direct determinant of HTF suitability but remains relevant for future scalability and techno-economic considerations, since low extraction efficiency may limit industrial competitiveness despite favorable thermophysical properties.

The acid value measured for the oil was 3.44 ± 0.02 mg KOH·g^–1^, indicating the presence of free fatty acids resulting from hydrolytic or oxidative degradation processes. The acid value which is slightly lower than values reported by Gonçalves^15^ (3.49 mg KOH·g^–1^) and substantially lower than those of Melo^16^ (12.24 mg of KOH/g), and (12.8 mg KOH·g^–1^) for Amaral.^13^ Because AV reflects the concentration of free fatty acids (FFAs) generated by hydrolysis and oxidative cleavage of triglycerides, lower values typically indicate better conservation status of the almonds and more controlled handling/extraction (e.g., minimized moisture/enzymatic activity and limited thermal/oxidative stress). In practical terms, a lower AV mitigates risks of corrosion in metal surfaces, soap formation during contact with alkaline media, and catalyst deactivation in downstream conditioning or upgrading steps—each of which can compromise the reliability of heat-transfer circuits and auxiliary operations.

Amaral^13^ cites a “standard” acidity on the order of ∼5 mg KOH·g^–1^ for acceptable-quality macaw oil, placing our value below that threshold and reinforcing its suitability for engineering evaluation. Conversely, elevated AV—as emphasized by Meher et al.^17^—often signals inadequate fruit selection, improper postharvest handling, or suboptimal extraction protocols, conditions that accelerate triglyceride degradation and can preclude certain applications due to saponification and deposit formation. For full comparability and process design, we report density (at 20 °C) and kinematic viscosity (at 40 °C) alongside AV, since density informs mass/heat balances and pump sizing, while viscosity governs pressure drop, heat-transfer coefficients, and start-up behavior; together with a low AV, these parameters provide a coherent basis for assessing oil stability and fit-for-purpose performance in thermal systems.

Building on Galvão,^18^ the lowest feasible acid values (AV) are preferred for vegetable oils because AV is a proxy for hydrolytic/oxidative alteration and, when elevated, can jeopardize downstream use (e.g., corrosion, soap formation, catalyst fouling). Reported AVs understandably span a range rather than a fixed point because they track (i) the degree of unsaturation (more unsaturation → greater oxidative susceptibility), (ii) seasonality and ecotype of the oilseed (climate and phenology shift lipid biosynthesis), and (iii) processing/storage history (drying, moisture control, time-to-extraction, oxygen/heat exposure). Consequently, literature values should be interpreted with these sources of variance in mind rather than as absolute pass/fail cutoffs.

It is important to note that vegetable oils inherently present higher acid values than engineered synthetic HTFs, and acid value alone should not be interpreted as a definitive exclusion criterion. However, for practical thermal-fluid deployment, pretreatment steps such as deacidification would likely be necessary.

The measured density was 0.917 ± 0.006 g·cm^–3^ at 20 °C, which lies within the range commonly reported for vegetable oils proposed for thermal applications.

Density directly affects thermal mass transport, pumping requirements, and volumetric heat-transfer considerations. The observed value suggests compatibility with general expectations for organic liquid thermal media.

While this measurement at 20 °C provides a reference physical characterization, comprehensive thermophysical mapping across the operating temperature range is envisioned for future thermal scaling and heat-exchanger design in medium-temperature solar systems.

The kinematic viscosity measured here (29.75 ± 0.76 mm^2^·s^–1^ at 40 °C) is lower than the value reported by Amaral^13^ (51.01 mm^2^·s^–1^ at 37.8 °C). Part of this discrepancy is consistent with the temperature dependence of viscosity—well described by Andrade/Arrhenius-type relations—wherein a modest increase from 37.8 to 40 °C reduces viscosity by enhancing molecular translational/rotational mobility and helping molecules surmount intermolecular interaction barriers.

Residual differences may reflect composition (e.g., higher lauric content tends to lower viscosity), FFA/water content, particle-size and extraction protocol, and analytical details (capillary constant, calibration). In practical terms, the combination of only slight density variation with a notably lower kinematic viscosity at 40 °C implies reduced pressure drop and improved pumpability at start-up, which are advantageous for heat-transfer service—if acidity remains controlled and oxidative stability is managed through appropriate conditioning.

The kinematic viscosity determined at 40 °C was substantially higher than typical synthetic HTFs.

Compared with highly viscous vegetable oils such as castor oil, this behavior is advantageous. However, compared with commercial engineered thermal fluids, viscosity remains a performance limitation requiring further assessment under operational temperatures.

Overall, the combination of moderate density and acceptable viscosity supports preliminary screening interest, although operational suitability remains dependent on dynamic thermal testing.

### 3.2 Fatty Acid Composition and Implications for Thermal Performance

Table 1 reports the fatty-acid composition of macaw almond oil, resolved by carbon-chain length, and juxtaposes these data with literature distributions for soybean and linseed oils.

**Table 1:** Composition in Fatty Acids (%), by Gas Chromatography, of Macaw Palm Oil Samples

	**Reference**	**This Work**	**Gonçalves** ^15^	**Amaral** ^13^	**Melo** ^16^	**Melo** ^19^
Fatty Acids	Abbreviated Formula	Macaw (%)	Macaw (%)	Macaw (%)	Soy (%)	Linseed (%)
Caprylic Acid	C8:0	2.98	3.92	5.22	-	-
Caproic Acid	C10:0	3.27	4.21	4.56	-	-
Lauric Acid	C12:0	39.36	45.15	44.14	-	-
Myristic Acid	C14:0	9.41	9.3	8.45	0.07	-
Palmitic Acid	C16:0	7.11	7.16	6.57	10.79	7.09
Palmitoleic Acid	C16:1	0.15	-	-	0.07	-
Stearic Acid	C18:0	2.99	2.25	2.11	4.23	6.53
Oleic Acid	C18:1	29.99	27.99	25.76	22.99	23.45
Linoleic Acid	C18:2	14.47	-	3.19	53.11	18.49
Linolenic Acid	C18:3	-	-	-	6.11	43.41
Arachidic Acid	C20:0	0.12	-	-	0.48	-
Gadoleic Acid	C20:1	0.14	-	-	0.17	-
Eicosadienoic Acid	C20:2	-	-	-	0.28	-
Eicosatrienoic Acid	C20:3	-	-	-	0.02	-
Erucic Acid	C22:1	-	-	-	0.33	-
Docosadenoid Acid	C22:2	-	-	-	-	-
Others		0.01	0.02	-	1.35	1.03
Total		100	100	100	100	100

Macaw palm almond oil is lauric-rich, with C12:0 ≈ 39%, and contains a substantial C18 unsaturated fraction—oleic (C18:1 ≈ 29.9–30%) and linoleic (C18:2 ≈ 14.5%)—accompanied by secondary saturates myristic (C14:0 ≈ 9.4%) and palmitic (C16:0 ≈ 7.1%). This blend of short/medium-chain saturates with C18 unsaturated aligns with prior reports for macaw oil^13,15^ and helps explain its fluid behavior: lauric/myristic fractions tend to lower viscosity and melting range, favoring pumpability at moderate temperatures, while oleic/linoleic contribute to oxidation susceptibility and thus inform the need for conditioning (e.g., antioxidants) in thermal applications.

In this data set, C18 species dominate macaw palm almond oil, with linoleic (C18:2 ≈ 53.1%) and oleic (C18:1 ≈ 23.0%) as the principal components, indicating a profile enriched in di- and monounsaturated. For lubricant uses, macaw palm almond oil can therefore be positioned as a C18-rich base stock that is promising when paired with antioxidant packages and, if needed, formulation strategies (e.g., blending with high-oleic oils or partial interesterification) to tune stability without sacrificing viscosity and film-forming performance.^15^

Factors such as alkyl-chain length, degree of unsaturation, and intermolecular interactions govern variations in viscosity. Unsaturated fatty acids predominantly occur in the cis configuration, introducing kinks that hinder close molecular packing; this reduces van der Waals cohesion and, consequently, lowers viscosity relative to analogous saturated chains of similar length.^19,20^

The significant lauric fraction observed here distinguishes macaw palm almond oil from many previously studied vegetable oils.

Potential engineering implications include advantages such as improved molecular packing stability; reduced oxidation sensitivity relative to highly polyunsaturated oils; lower viscosity compared with long-chain saturated oils. And limitations include: remaining unsaturated fractions still permit oxidative degradation; thermal polymerization risk remains relevant.

Compared with soybean and linseed oils, macaw palm oil contains lower extreme polyunsaturation, which is favorable for thermal stability.

Compared with highly saturated oils, the presence of oleic and linoleic acids may improve fluidity.

This mixed composition suggests an intermediate balance between thermal robustness and flow behavior, which justifies further HTF evaluation.

However, fatty acid composition alone cannot establish operational suitability and should be interpreted only as a first-order indicator.

### 3.3 FTIR Analysis

Functional groups were qualitatively identified by FTIR spectroscopy. The resulting spectra (Figure 1) displays characteristic vibrational bands whose positions and intensities reflect the molecular environments of the functional groups present in the sample. The FTIR spectrum (600–4000 cm^–1^) exhibits the expected intense bands for triglyceride-rich vegetable oils. Consistent with Melo^16^ prominent absorptions at ∼2925 and ∼2851 cm^–1^ correspond to the asymmetric and symmetric C–H stretching of methylene/methyl groups (−CH_2_/–CH_3_) in free fatty acids and triacylglycerols, reflecting saturated sp^3^ hydrocarbon chains. A strong band near ∼1747 cm^–1^ is diagnostic of the ester carbonyl stretching (ν­(C=O)), confirming the triglyceride backbone; weaker features typically appear around 1465/1377 cm^–1^ (CH_2_/CH_3_ bending) and ∼1160–1050 cm^–1^ (C–O stretching of the ester linkage), with a ∼720 cm^–1^ rocking band indicative of long-chain methylenes. Together, these assignments support a lipid matrix dominated by triglycerides with the usual aliphatic signatures.^21^

**Figure 1:**
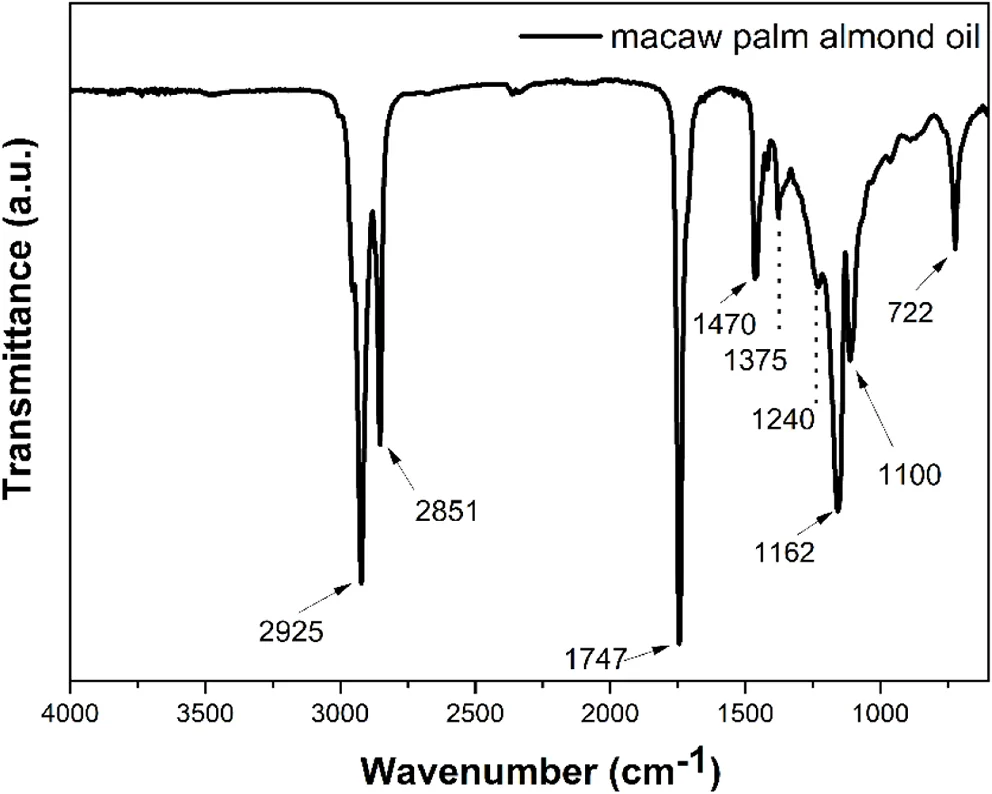
Absorption spectrum in the infrared region for a macaw palm almond oil sample.

The characteristic band did not occur at ∼1710 cm^–1^, a factor associated with the −C=O deformation of carboxylic acids,^16^ indicating a low level of free fatty acids present in the analyzed oil.^22^ Although titrimetric acid value confirms measurable free fatty acid presence, this apparent discrepancy is not unusual, since FTIR sensitivity differs from titrimetric quantification. These spectra help to evaluate the deoxygenation content of products formed due to the presence of oxygenated groups. The bands at 1470 and 1375 cm^–1^ represent angular deformation of sp^3^ carbons C–H bonds stretching.^22^ The stretching vibrations of the 1240 and 1162 cm^–1^ bands regard the stretching of the −C–O ester bonds.^16^

A prominent band near ∼1100 cm^–1^ is assigned to the C–O stretching of the ester linkage in triacylglycerols.^16^ In addition, the feature at ∼722 cm^–1^ corresponds to the methylene rocking mode of −(CH_2_)_n_– segments (with *n* > 3), evidencing long aliphatic chains and thus the presence of fatty acyl moieties typical of triglyceride-rich oils.^21,22^

Importantly, FTIR serves here as qualitative structural confirmation rather than direct operational HTF validation.

### 3.4 UV–Visible Analysis

UV–Vis spectroscopy (Figure 2) shows that changes occurred in the radiation absorption of macaw palm almond oil during the compound’s degradation process. The absorptions near 232 and ∼270 nm indicate formation of conjugated dienes/trienes during early and advanced oxidation, respectively; signals remain modest in the 300–400 nm window, consistent with limited secondary chromophores within the tested conditions.^23,24^ Thus, each wavelength relates to changes in the structures of triacylglycerols or the formation of new molecules.^23^

**Figure 2:**
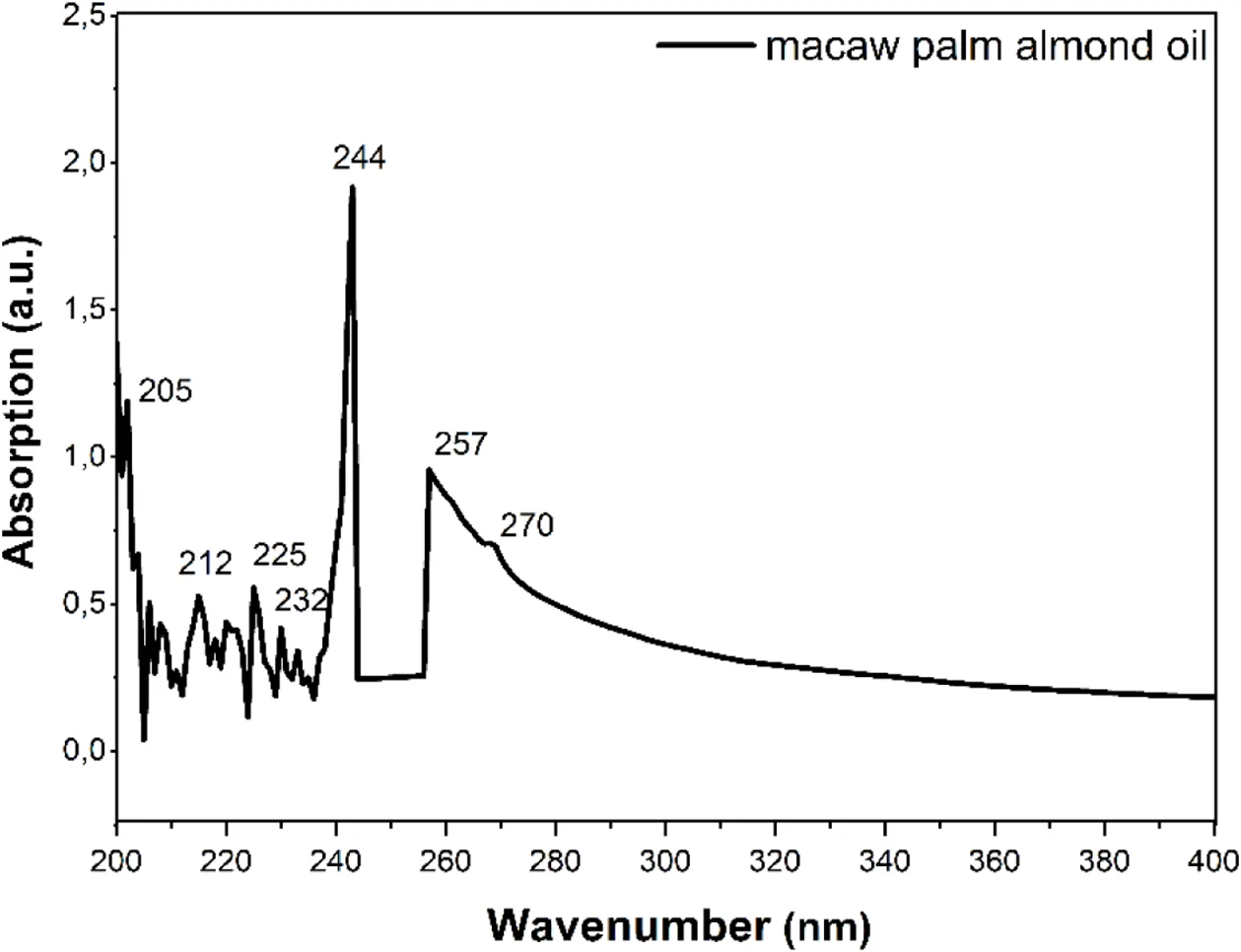
UV–Vis absorption spectra of a macaw palm almond oil sample.

The UV–Vis spectrum displays absorption in the 200–244 nm region, attributable to primary oxidation products of oleic, linoleic, and linolenic acids—i.e., conjugated dienes exhibiting π → π* transitions—along with a secondary envelope at ∼257–270 nm associated with oxidized species (e.g., aldehydes, oligomers/dimers) and conjugated trienes, also via π → π* transitions; the dominant maximum occurs at ∼244 nm. Oliveira^23^ also found this relationship. Besides that, the presence of small noises, with a large shift toward longer wavelengths, due to the large number of conjugated doublets, makes the higher and lower energy orbitals come closer together.^25^

Because unsaturated fatty acids are abundant in macaw palm almond oil, it is inherently more susceptible to oxidative and thermal degradation than highly saturated oils; double bonds are reactive sites that facilitate peroxide formation and subsequent chain-scission/polymerization pathways. Even so, endogenous antioxidants (e.g., phenolics/tocopherols) can delay the onset of oxidation and slow compositional change, helping preserve the initial profile during early stages of thermal exposure—they mitigate but do not prevent degradation, so susceptibility ultimately increases as antioxidants are depleted.

No discernible absorption was observed in the 300–400 nm region, which suggests a low concentration of secondary oxidation chromophores (e.g., conjugated polymers, carbonyl–dienyl species) rather than a simple paucity of unsaturated fatty acids.^23^ This spectral quietness is consistent with limited photooxidative progression under the tested conditions and indicates that the triacylglycerol double bonds remain largely unconverted to strongly absorbing secondary products, i.e., the unsaturation is still largely intact despite early stage primary oxidation signals at shorter wavelengths.

Absorption features observed are compatible with conjugated molecular structures associated with unsaturated lipid systems.

The presence of early oxidation markers suggests some susceptibility to oxidative processes, consistent with the unsaturated fatty acid content.

Overall, the presence of primary (232 nm) and secondary (∼270 nm) oxidation markers directly reflects the chemical stability of the fluid under thermal stress. Thus, UV–Vis profiling is essential in heat-transfer fluid (HTF) applications, as thermo-oxidative degradation directly impairs system operation. As a result, the accumulation of primary peroxides and secondary products generates free fatty acids, fouling, and increased viscosity.

Therefore, UV–Vis findings reinforce the need for oxidative aging evaluation before practical implementation.

From an engineering perspective, mitigation of this oxidative susceptibility could involve controlled addition of phenolic antioxidants to inhibit radical-chain oxidation, together with oxygen-exclusion strategies such as nitrogen blanketing of storage and expansion vessels and minimization of air ingress into the circulating loop. Metal passivators may also reduce catalytic oxidation promoted by contact with metallic surfaces, while blending with more oxidation-stable fluids represents another possible formulation strategy. The type and concentration of antioxidant additives should, however, be optimized through accelerated oxidative-aging and thermal-cycling experiments, together with compatibility tests involving seals and construction materials, before implementation in a practical HTF system.

### 3.5 Thermal Stability (TGA)

Figure 3 displays the thermogravimetric (TGA) profile, which reveals a multistage mass-loss behavior rather than “exothermic events” (those are captured by DSC). The oil shows an onset of measurable volatilization/decomposition with ∼10% mass loss at ∼300 °C, followed by a major degradation stage up to ∼480 °C that accounts for ≈95% of the initial mass, consistent with thermal cleavage and volatilization of triglyceride fragments. Above ∼480 °C, the residual ∼5% decreases only slowly, which is attributable to the thermal decomposition of polymerized/refractory residues formed during heating. Collectively, the TGA trace indicates limited thermal stability above ∼300 °C and a dominant decomposition window between ∼350–480 °C under the stated experimental conditions.

**Figure 3:**
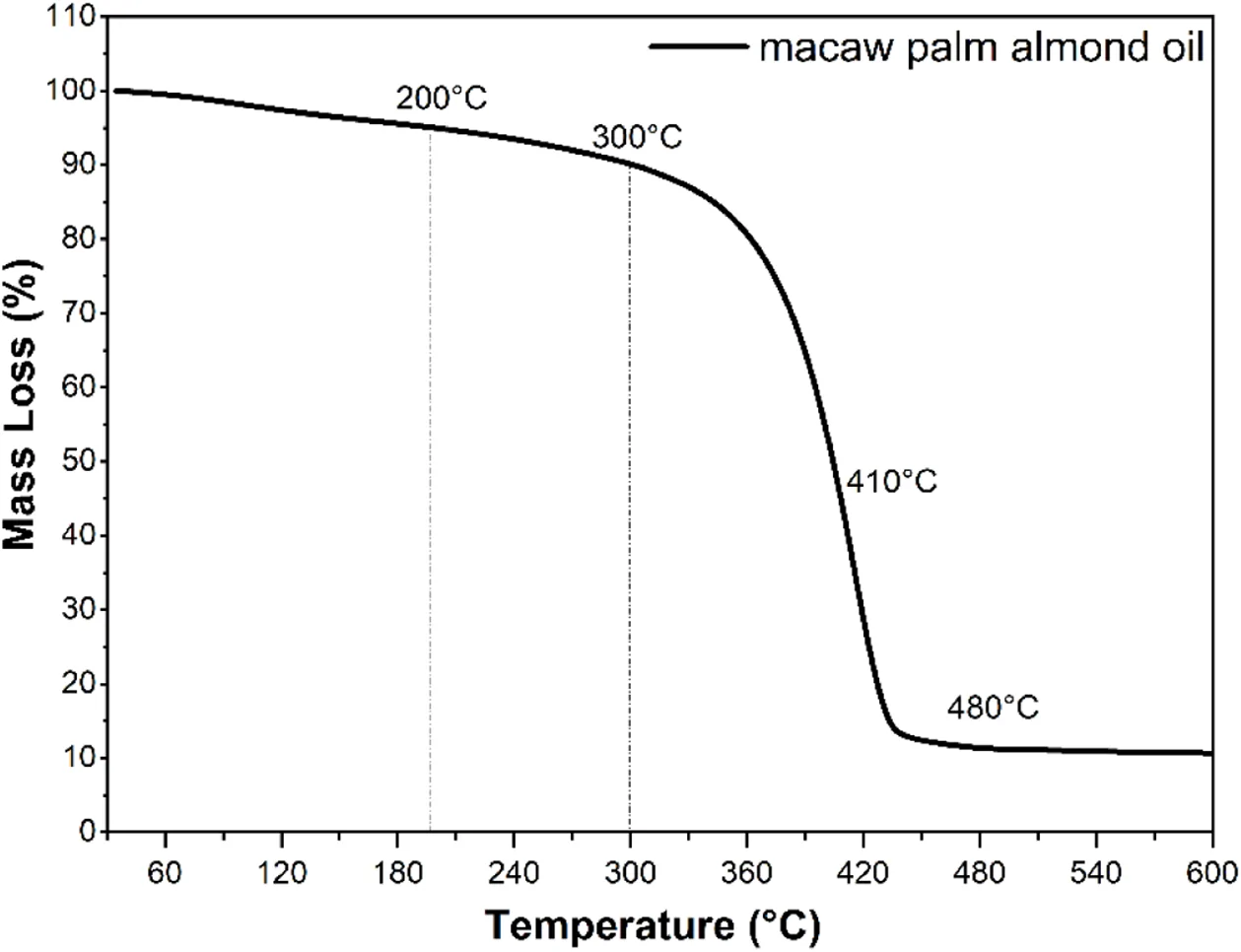
TGA curve for a macaw palm almond oil sample at a rate of 20 °C/min.

Accordingly, the primary deoxygenation and cracking of macaw palm almond oil are inferred to occur predominantly within ∼350–450 °C, consistent with the TGA major-loss window observed here and with literature reports that place triglyceride thermal fragmentation events broadly between ∼235 and 490 °C.^16,26,27^ Mechanistically, this range encompasses acylglycerol bond scission, β-scission of alkoxy/alkyl radicals, and decarboxylation/decarbonylation pathways that yield lighter hydrocarbons and oxygenates, followed by secondary volatilization and slow decomposition of polymerized residues at higher temperatures.

In soybean oil, Melo^16^ reported a single main decomposition stage spanning ∼340–480 °C, with the maximum degradation rate near ∼415 °C, and no evident inflection in the TGA trace prior to that stage. By contrast, the macaw palm almond oil examined here shows an early inflection around ∼200 °C, plausibly reflecting moisture release and volatilization of free fatty acids (≈5%) before the onset of the primary mass-loss event, a predecomposition shoulder that is absent in the soybean oil profile.

This result suggests that macaw palm almond oil may possess sufficient short-term thermal resistance for intermediate temperature applications (250–300 °C) represents the ideal operating window for macaúba kernel oil as a heat transfer fluid.

However, an important engineering distinction must be emphasized: thermal decomposition onset is different from the operational HTF qualification.

TGA indicates decomposition behavior under controlled laboratory heating conditions, but does not capture: oxidative aging; repeated thermal cycling; long-term residence effects; flow-induced degradation; interaction with engineering materials.

Thus, while TGA supports preliminary interest, it cannot independently validate HTF suitability.

### 3.6 Differential Scanning Calorimetry (DSC)

At a heating rate of 10 °C·min^–1^, the DSC trace shown in Figure 4 exhibits a series of thermal events between approximately 25 and 220 °C. A low-temperature feature near ∼25 °C (≈0.40 W·g^–1^, according to the instrumental convention adopted) may be associated with the release of residual moisture and/or low-molecular-weight volatile compounds, corresponding qualitatively to the initial shoulder observed in the TGA profile. This event is followed by a distinct endothermic transition around ∼27 °C (≈−0.39 W·g^–1^), which is attributed to the melting of triacylglycerol crystalline fractions commonly observed in vegetable oils.

**Figure 4:**
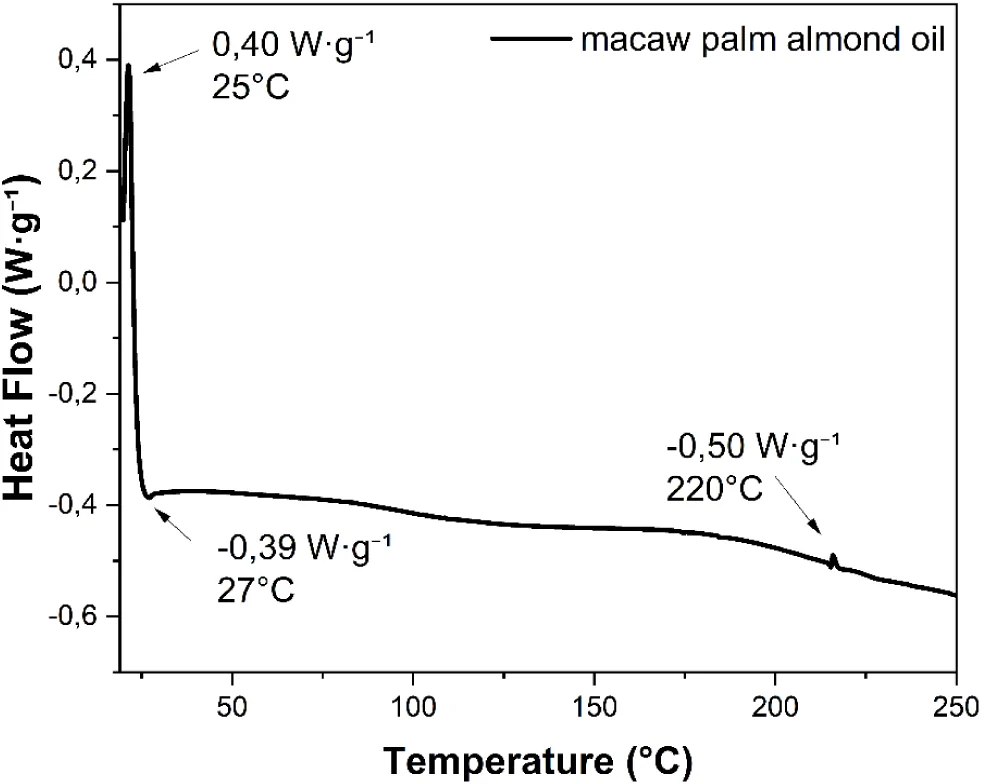
DSC curve for macaw palm almond oil sample.

A broader endothermic process extending up to ∼220 °C (peak signal ≈ −0.50 W·g^–1^) is associated with volatilization phenomena and the onset of thermal degradation, in qualitative agreement with the initial mass-loss stage observed in the TGA curve.

Carvalho^28^ states that the composition of fatty acids in vegetable oils is directly linked to decomposition enthalpies. In this context, the thermal behavior observed here may be related to the presence of volatile compounds generated during the degradation of unsaturated triacylglycerol fractions, with contributions from monounsaturated and saturated fatty acids.^29^

Fonseca^29^ investigated the thermal stability of crude buriti and macaúba oils by DSC and reported higher enthalpic events for buriti oil (58.68 J·g^–1^) compared with macaúba oil (0.97 J·g^–1^), suggesting differences in lipid organization and thermo-oxidative behavior between these vegetable oils. Similarly, the macaw palm almond oil evaluated in the present study exhibited a relatively stable thermal profile and the absence of pronounced exothermic oxidative events throughout the analyzed temperature range.

A more resolved interpretation of the DSC data would benefit from repeated scans and complementary thermal analyses—for example, modulated DSC, variable heating rates, isothermal holds, and parallel TGA under inert vs oxidative atmospheres—to deconvolute overlapping events, refine baseline corrections, quantify enthalpies with greater confidence, and better distinguish moisture release, melting, and early volatilization/decomposition contributions.

DSC provides useful thermal behavior information but should not be interpreted as direct evidence of operational heat-transfer performance.

### 3.7 Comparative Engineering Assessment for HTF Applications

The heat-transfer fluid (HTF) is pivotal to CSP performance because its allowable bulk operating temperature and transport properties govern thermal efficiency, pressure drop, start-up behavior, and materials compatibility; raising HTF temperature generally improves cycle efficiency (within stability and safety limits). Accordingly, Table 2 compiles the thermophysical/chemical properties measured for macaw palm almond oil alongside those of commonly used HTFs in solar-thermal systems to provide an application-oriented benchmark. For transparent comparison, we report density (at 20 °C) and kinematic viscosity (at 40 °C) for macaw palm almond oil and list the reference temperatures and property types for literature fluids (e.g., dynamic vs kinematic viscosity, elevated-temperature data for silicone oils, VP-1, and molten salts). Because viscosity is strongly temperature-dependent and some references provide dynamic rather than kinematic values, cross-fluid numerical contrasts should be read as qualitative unless normalized to a common temperature and property definition. Within these caveats, the table situates macaw palm almond oil relative to incumbent fluids in terms of pumpability, thermal window, and conditioning needs (e.g., deacidification, antioxidant/corrosion-inhibitor packages) for medium-temperature CSP service.

**Table 2:** Thermophysical-Chemical Properties of Macaw Oil and Other Types of Vegetable, Synthetic and Mineral Oils

Parameter	Vignarooban et al.^2^	Vignarooban et al.^2^	This Work	Gomna et al.^7^	Vignarooban et al.^2^	Gomna et al.^7^	Vignarooban et al.^2^
Fluid	Air	Water	Vegetable	Vegetable	Synthetic	Synthetic	Mineral
Oil	-	-	Macaw palm almond	Jatropha curcas oil (Jatropha nut)	Therminol VP-1	Silicone	Solar salt NaNO_3_ (60%)–KNO_3_ (40%)
Acidity Index (mg of KOH/g)	-	-	3.44	0.04	0.03	0.01	0.015–1.2
Dynamic Viscosity	0.03.10^–3^ Pa.s (600 °C)	1.33.10^–3^ Pa.s (600 °C)	-	-	0.6.10^–3^ Pa.s (300 °C)		2.10^–3^ Pa.s (500 °C)
Kinematic Viscosity	-	-	29.75 (mm^2^/s) at 40 °C	39.72 (mm^2^/s) at 40 °C	-	35–40 (mm^2^/s) at 40 °C	-
Density 20 °C (g/cm^3^)	0.001	0.998	0.917	0.912		0.96–1.1	
Specific heat capacity (J/g·K)	1.12 (600 °C)	2.42 (600 °C)	-	1.92	-	1.5	1.1 (600 °C)
Thermal conductivity (W/m·K)	0.06	0.08	-	-	0.1	0.15	0.55
Operating temperature (°C)	700	<400	<300 (10% loss)		393		550
Melting temperature (°C)	-	0	27	35	<15	<20	223
Oxidative stability	Great	Great	Susceptible to oxidation	Susceptible to oxidation	Great	Great	Good

Relative to highly viscous oils (e.g., castor), flow behavior appears more favorable.

Relative to conventional engineered HTFs, important limitations remain: higher viscosity; uncertain oxidation lifetime; acid value concerns; unknown corrosion compatibility; lack of validated heat-transfer coefficient data.

Crude macaw palm almond oil presented a high acidity value (3.44 mg KOH/g) of synthetic and mineral oils (molten salts) used in a CSP system, possibly causing corrosion of metal parts of the absorber tubes because of the formation of free fatty acids.^16^ Amaral^13^ discloses that the acidity level is directly related to oil conservation. To reduce acidity, it is possible to apply the deacidification process of this oil. Given this, obtaining a better-quality product depends on greater control over the collection, handling, and storage processes of macaw fruit, as well as the oil extraction stage.

Viscosity is a key determinant of CSP viability because it governs mass flow, pressure drop, pump power, and start-up behavior; since viscosity falls sharply with temperature, lower-viscosity HTFs generally enable better transport conditions within a given piping layout. In our measurements, macaw oil exhibits a kinematic viscosity of 29.75 mm^2^·s^–1^ at 40 °C, which is lower than values cited for silicone oils (≈35–40 mm^2^·s^–1^ at 40 °C) and jatropha oil (≈39.72 mm^2^·s^–1^ at 40 °C). For context, literature often reports dynamic viscosities at elevated temperatures for incumbent HTFs (e.g., Therminol VP-1 at ∼300 °C; solar salt at ∼600 °C), so direct numerical ratios can be misleading without normalization to the same temperature and property type (kinematic vs dynamic). With that caveat, the comparison still positions macaw oil as relatively pumpable at moderate temperatures, suggesting reduced pressure losses versus higher-viscosity alternatives at equal conditions, while emphasizing that meaningful cross-fluid benchmarking must account for temperature-dependent viscosity curves and the kinematic/dynamic distinction. It should be noted that viscosity was experimentally determined at a single reference temperature of 40 °C in the present study. Therefore, a complete viscosity–temperature relationship cannot be established from the current data set. Nevertheless, the measured value provides a useful preliminary indication of the oil transport behavior. Since viscosity strongly influences pressure losses and pumping energy requirements, future studies should evaluate this property over the intended operating temperature range to support hydraulic design, circulation modeling, and more accurate assessment of pumping requirements in heat-transfer systems.

Fluids with high density and thermal capacity values are the most suitable for working HTF and for thermal storage, as they increase the system’s efficiency. Macaw palm almond oil density was 0.917 g/cm^3^ at 20 °C, consistent with thermal fluids presented in Table 2. The calorific capacity of macaw palm almond oil was not determined in this work. However, Amaral^13^ verified that macaw almond oil’s calorific value was 4739 kcal/kg, revealing the high calorific value of the species in comparison to other oilseeds such as soybean and sunflower oil (2200 and 1700 kcal/kg, respectively).

Thermal conductivity and maximum operating temperature are pivotal HTF parameters because they directly control heat pickup within absorber tubes and subsequent heat exchange (typically to high-pressure water/steam). Higher thermal conductivity improves the internal heat-transfer coefficient and reduces temperature gradients, while a higher allowable bulk temperature enables greater thermodynamic efficiency—provided the fluid remains chemically and thermally stable (no cracking, oxidation, or excessive vapor pressure) and materials-compatible. Together, these properties largely determine HTF’s suitability for service in CSP loops and should be considered alongside viscosity, specific heat, and corrosion risk when selecting fluids for plant operation.

Hoffman et al.^12^ state that increasing temperature can reduce thermal conductivity. This work could not determine the thermal conductivity of macaw oil because of limitations in the reproducibility of the analyses. Pure macaw palm almond vegetable oil had an irregular operating temperature at temperatures <330 °C with a loss of 10% of its initial mass, a lower condition than other fluids commonly applied in CSP systems. However, its temperature exceeds the temperatures obtained, for example, in plants with a linear Fresnel reflector where they reach a temperature of 250 °C.

A more in-depth investigation is required to achieve this condition, especially concerning low lower-limit temperatures and high upper-temperature limits at low pressures, aiming at safety and greater efficiency in the conversion of heat into electricity in the system. Halogenated salts can operate at high temperatures (<1000 °C), but they have a high melting point, making their application unfeasible.^12^ Such factors can also be seen in other HTFs.

The melting temperature is another important factor for verifying the technical-economic viability of the system. Despite the high melting temperature of macaw oil (Table 2), it was lower than the temperature of molten salts (between 65 and 400 °C). Solar salt has a melting and operating temperature of 223 and 550 °C, respectively, which raises freezing/solidification concerns. This fact increases the cost of the plant due to the need for protection systems against fluid solidification in solar field absorber tubes and thermal storage tanks.

Regarding the oxidative stability of macaw oil, two stages of chemical degradation of deoxygenation and cracking were identified, relating events of thermal fragmentation reactions of triglycerides between 235 and 490 °C. Therefore, this sensitivity can alter chemical and physical properties compared to other thermal fluids. The use of corrosion-inhibiting additives and antiwear agents can benefit in controlling oil mass degradation caused by thermal stress. Therefore, a cautious investigation of its oxidation stability and thermal aging is recommended for further conclusions for solar applications.

In this sense, the applied experiments expand and strengthen the benefits of macaw palm almond oil in the chain of oils commonly used in solar power plants, partially replacing synthetic and mineral oils, and increasing environmental gains as they are renewable and biodegradable resources. Engineering implications: deploying macaw oil as HTF in CSP requires (i) deacidification to curb corrosion risk, (ii) antioxidant packages to delay oxidation under cyclic heating, and (iii) materials compatibility checks (seals, elastomers, steel alloys). Phenolic antioxidants and passivators can extend oil lifetime at 250–320 °C; blending with higher-stability fluids is another pathway to tailor viscosity and oxidative resilience without process redesign.

Therefore, the present findings support classification of macaw palm almond oil as a promising renewable screening candidate, not a validated HTF replacement.

### 3.8 Engineering Feasibility, Limitations, and Future Perspectives for HTF Application

The results indicate that macaw palm almond oil is a promising renewable candidate for medium-temperature heat-transfer fluid (HTF) applications due to its favorable physicochemical and thermal properties, renewable origin, biodegradability, and potential regional availability in Brazil. Preliminary density, viscosity, fatty acid composition, and thermogravimetric behavior support its initial feasibility.

However, this study represents only a preliminary screening, not operational HTF validation. Key engineering aspects remain unresolved, including long-term oxidative stability, thermal aging, thermal cycling, corrosion compatibility, heat transfer efficiency, deposit formation, and real circulation performance. Although the acid value was lower than some literature reports, further fluid conditioning may still be required. Additionally, TGA stability under laboratory conditions should not be directly interpreted as safe operational performance.

Future work should focus on thermal aging, corrosion testing, pilot-scale validation, heat transfer characterization, CFD simulation, techno-economic analysis, lifecycle assessment, and fluid stabilization strategies. Overall, macaw palm almond oil shows potential as a sustainable HTF, but requires further engineering validation before practical application.

## 4 Conclusions

Although several vegetable oils (soybean, jatropha, palm, rapeseed) have been investigated as biobased HTF candidates, no previous study has specifically reported the thermophysical screening evaluation of macaw palm almond oil for this application.

Macaw palm almond oil (*Acrocomia aculeata*) was evaluated through physicochemical and thermophysical characterization as a potential renewable heat-transfer fluid (HTF) candidate for medium-temperature solar thermal applications, with a practical operating limit below 300 °C to prevent thermal breakdown, volatilization, and deposit formation under real-world system conditions.

The oil exhibited an extraction yield of 15.74%, acid value of 3.44 mg KOH·g^–1^, density of 0.917 g·cm^–3^ at 20 °C, and kinematic viscosity of 29.75 mm^2^·s^–1^ at 40 °C. Its fatty acid profile, dominated by lauric, oleic, and linoleic acids, together with FTIR, UV–Vis, and thermal analyses, indicates physicochemical characteristics compatible with preliminary HTF screening. However, this study represents a first-stage screening assessment rather than operational HTF validation. Critical issues such as long-term oxidative stability, thermal cycling performance, corrosion compatibility, heat transfer efficiency, circulation behavior, and techno-economic feasibility remain unresolved. In addition, the observed acid value and susceptibility to oxidation suggest that fluid conditioning or pretreatment may be required.

Despite these limitations, macaw palm almond oil offers sustainability-related advantages, including renewable sourcing and biodegradability, expanding the portfolio of biobased candidate materials for solar thermal applications. Future studies should focus on accelerated aging, thermal performance validation, corrosion testing, process simulation, lifecycle assessment, and pilot-scale implementation.

## Data Availability

The data sets generated and analyzed during the current study are available from the corresponding author upon reasonable request.
